# Current state of dynamic surgery. A literature review

**DOI:** 10.4317/medoral.24566

**Published:** 2021-05-23

**Authors:** Anna Parra-Tresserra, Jordi Marquès-Guasch, Jordi Ortega-Martínez, Joan Basilio-Monné, Federico Hernández-Alfaro

**Affiliations:** 1 DDS, PhD Student, School of Dentistry, Universitat Internacional de Catalunya, Barcelona, Spain; 2 DDS, PhD Student, Assistant Professor, School of Dentistry, Universitat Internacional de Catalunya, Barcelona, Spain; 3 DDS, PhD, Assistant Professor, School of Dentistry, Universitat Internacional de Catalunya, Barcelona, Spain; 4 MD, PhD, Professor and Head of Department of Integrated Dentistry Department, School of Dentistry, Universitat Internacional de Catalunya, Barcelona, Spain; 5 MD, DDS, PhD OMFS, Professor And Head of Department of Medicine, Surgery and Oral Implantology Department, School of Dentistry, Universitat Internacional de Catalunya, Barcelona, Spain

## Abstract

**Background:**

Recently, dental implant technology has been widely used for oral reconstruction. Dental implants are the treatment of choice for those patients with dental absences. An optimal implant placement is based on the prosthetic driven concept in order to achieve an aesthetic and functional restoration with a long-term prognosis. There are two types of guided implant surgery that are described in the literature: Static Guided Surgery (SGS) and Dynamic Guided Surgery (DGS). The aim of this study is to be aware of the current state of dynamic surgery and compare in the literature the discrepancies between planning and placement of dental implants.

**Material and Methods:**

The study consists of a bibliographic review on the topic. The research has been performed in the Medline/Pubmed of articles published by different professional associations and societies in the international context.

**Results:**

Twenty two studies out of 100 articles from the initial search were finally included. Our results have been compared with other current available papers in the literature reviewed that obtained similar outcomes.

**Conclusions:**

Dynamic navigation shows a better accuracy and precision of implant placement. To corroborate the results of this review as well as to evaluate the different variables that could influence the accuracy of this technique, future randomized control trials will be needed.

** Key words:**Guided surgery, dynamic navigation, dynamic guided surgery, computer assisted surgery.

## Introduction

In the recent years, dental implant technology has been widely used for oral reconstruction. Dental implants are the treatment of choice for those patients with dental absences. A dental implant prothesis will reestablish chewing function, has higher biomechanics and aesthetics than conventional prosthesis, and facilitates efficient long-term care (1).

- Prosthetic driven concept

An optimal implant placement is based on the prosthetic driven concept in order to achieve an aesthetic and functional restoration with a long-term prognosis (1,2).

It is widely known that the accuracy of 2-dimensional images fail to achieve the information needed for dental implantation. In contrast to what computed tomography can provide, images in the 3-dimensional space through softwares of data reorganization (1). The combination between 3D images and surgical planning software allows to visualize the prosthetic outcome together with the anatomical structures reducing then the risk of a iatrogenic lesion and improving the communication between the surgeon, the prosthodontist and the patient (1-3).

In a brief summary, having an implant positioned in the correct 3D position will give dentists many advantages: favorable aesthetic and prosthetic outcomes, long-term stability of peri-implant soft and hard tissue due to simplifying the hygiene and improving the occlusal loading. Furthermore, an implant placed in the correct position allows the final prostheses to be excellent designed and enables to arrange and fabricate retrievable screw-retained supra-estructures, by that evading non-retrievable cemented restorations (4,5).

Therefore all of these advantages contribute to the long-term success of dental implants. Desired inter-implant distance, tooth-to-implant distance, implant depth are some of the requirements that have made implant planning an important appliance when regarding for optimal success. Besides it will also enable an excellent communication between dentists and patients (6,7).

There are many methods nowadays for implant placement such as freehand approach, guided surgery with hand made guides at the laboratory, and finally, the most recent method, computer-aided design/computer-aided manufacturing (CAD/CAM) - generated static guides that can be either tooth, mucosa or bone supported (6).

- Computer- Assisted Surgery

Three-dimensional dental software was introduced in 1988 by Columbia Scientific, inc (Glen, burnie, MD, USA) and transformed computerized tomography axial slices into reformatted cross-sectional images of the alveolar ridges in order to diagnose and evaluate (5).

In implant dentistry the term “guided surgery” can be defined as a digital workflow starting with collecting the patients data for the future prothesis. Afterwords the data is processed through a virtual planning software and finally a stereolithographic template is produced via a prototyping system. The most known prototyping technique and more frequently used in dentistry for producing surgical guides via CAD/CAM system is the stereolithography (8).

It has been proved that guided surgery is a confidence-building and accurate method, that reduces the risk of damaging the alveolar nerve, sinus perforation, fenestration and dehiscence (8,9).

There are two types of guided implant surgery that are described in the literature: Static Guided Surgery (SGS) and Dynamic Guided Surgery (DGS). The first one, refers to the use of static surgical template (5). A costume drilling guide is digitally designed as part of the planning process and manufactured prior to the surgery. During the surgery the guide is placed on the patients jaw, mucosa or teeth and the metal sleeves are used to guide the drilling process prior to the implant insertion (10). This will reproduce the virtual implant position directly from computerized tomographic data to a surgical guide, which prevent any intra-operative change of the implant position (5).

The dynamic approach can be defined as a real-time coordination of the surgeon's hands and eyes by 3-dimensional visualization of the implant preparation with high magnification (6). It provides real-time guidance to the surgeon, who is operating freehand. The navigation system will track the position of the tip of the drill and will map it to a pre-acquired CBCT scan of the jaw to allow a real-time drilling and placement guidance. Once the drill approaches a pre-planned implant location, the system gives a cross-hair display to guide the surgeon to accurately locate the drill tip at the entry point planned, settle the drill orientation to the planned entry angle and to drill the planned depth. One time the osteotomy preparation is complete, the same approach can be used to guide the implant insertion. This will show differences between the drill tip’s position, angulation and depth comparing it with its virtually planned position, angulation and depth (10).

Despite dynamic surgery requieres an upfront investment in training and work, it has the potential to provide many advantages over the static approach (10):

1. It allows to have in a single appointment: scanning, planning and surgery. (When a CBCT is available on site) (10).

2. Safety and predictability are increased due to the ability of verifying accuracy at any time during the surgery (10).

3. The planning is simpler and faster, surface segmentation and guide design has not to be printed and can be made at the clinic (10).

4. The pre-procedure costs are apparently lower (10).

5. Ergonomics of the surgeon are improved (10).

6. Failure of guidances are eliminated because there is not fractured or badly fitted guides (10).

- Accuracy considerations

Implant placement and depth control is more accurate when using CT-generated guide stents compared to freehand procedures or model-based nonrestricted guides. Block *et al*. [2017] assert that half of the implants placed with static guides are placed more superficially than planned (6). The main performance characteristic of Computer Assisted surgery is accuracy. Guidance offers obvious accuracy advantages (11). Comparing static and dynamic navigation accuracy has recently been studied and most of the studies found in the literature are *in vitro* (10,12).

In order to evaluate accuracy of implant position, preoperative and postoperative CBCT is employed and used for measuring and comparing entry point and apex deviation as well as differences between guided and non guided implant placement. There are not many studies to date measuring *in vivo* placement accuracy obtained with the use of navigation system (10). In other words, *in vivo* accuracy has never been properly studied and documented (11,13,14).

When using dynamic navigation changes, if wished, can be made at the time of the surgery, including implant size, length, width, shape and changes in positioning as required clinically (15,16).

It is not known if implant placement accuracy is truly higher when using navigation and it will be necessary in the near future that accuracy and precision is established in all navigation systems (15).

- Navigation system accuracy

As Block *et al*. [2017] indicate in their study, dynamic navigation systems have a mean entry deviation approximating 0,4mm and mean angular deviation error approximating 4 degrees. Implant placement accuracy in all the studies simulating dynamic navigation is very variable and for this reason it was decided to carry out the review (6,17).

The aim of this study is to be aware if the current state of dynamic surgery and compare in the literature the discrepancies between planning and placement of dental implants.

Dynamic navigation is relatively new to dental implant placement and there is a need to know if a true learning curve has to be achieved in order to be a proficiency clinician (6).

## Material and Methods

The study consists of a bibliographic review on the topic, Current state of dynamic surgery, and a comparison between the implant planned position and the clinical implant position. To identify studies for the purpose of this review, the definition of the PICO question (population, intervention, comparison, outcome) was used to guide construction of the search strategy: *P* = patients receiving dental implant placement surgery; I = dynamic guided implant placement surgery; C = comparison between planned implant position and real clinical position; O = accuracy evaluation based on mean horizontal deviation, mean vertical deviation, mean vertical deviation, and mean angle deviation). The question posed in the review was: Is Dynamic Guided Surgery an accurate system for placing dental implants?

- Search Strategy

An electronic search was conducted in the National Library of Medicine (Medline via PubMed) Combination of controlled terms and key search terms were used. Including: “guided surgery”, “dynamic navigation”, “dynamic guided surgery”, “computer assisted surgery”

- Inclusion criteria

Prior to reading the retrieved abstracts, consensus was reached on criteria to be applied for further full text evaluation. For evaluation of clinical performance:

1. Study population of at least 10 patients undergoing dynamic guided surgery

2. Observational studies

3. Randomized controlled trials

4. Comparative studies

5. In vitro studies of at least 20 samples

6. Evaluation of surgical complications during implant insertion

7. Outcomes of the surgical intervention and immediate postoperative period

8. Differences between the implant planned position and the clinical implant position

Articles were excluded after full-text analysis when not reporting on the above listed outcome variables and case reports including less than 10 subjects.

- Data collection and analyses

One reviewer extracted the data and it has been included only if it accorded to the criteria mentioned above. The following parameters were evaluated: global deviation at the entry point (mm), global deviation at the apex of the implant (mm), and angular deviation: degrees, differences between static and dynamic guided surgery. Finally, a discussion has been elaborated comparing the results obtained in the different studies and the content of each of the articles included in this study in order to reach a final conclusion.

## Results

Twenty two studies out of 100 articles from the initial search were finally included. Our results have been compared with other current available papers in the literature reviewed that obtained similar outcomes.

## Discussion

When placing implants, accuracy is essential in order to provide the best care of patients. If the accuracy is not reached, implants can still be restorable in many cases but will requiere additional costs for the dentist and patient: use of abutments, angled screws, deeper cement margins, increased chair time (6-15).

Development of navigated surgery have permitted an understanding of the various levels of guidance (fully guided, partially guided and freehand) (6-15).

Freehand placement compared with guided methods was less accurate. Dynamic guided system is an improvement over freehand placement and accuracy is similar to the obtained with static guides. Moreover, it is not known if dynamic navigation will improve accuracy and precision of implant placement (6-15).

A large number of authors agree that dynamic navigation has advantages over static navigation: allowing real time modifications of the surgical plan and a direct visualization of the surgical field is always allowed. The drill site visualization is not interfered by the static guide, it can be used on patients with limited mouth opening, the dynamic guide is not restricted by the drill tube size so narrow single-tooth situations can be completely guided.

Systems of dynamic navigation are absolutely “open” and do not have the need for special instrumentation. There is a lack of need to fabricate a 3D printed guide Dynamic navigation allows the surgeon an improvement of ergonomics. Furthermore the surgeon visualizes the surgical field during the drilling procedure while looking at a computer monitor and does not need to bend over or twist to place the implant (5-15).

- Sources of guidance and placement errors

Common reason for error when placing implants: deviation of the implant position due to asymmetric density in bone. Dense bone will deflect drills when osteotomies are created and implants are delivered (6,10-12,15).

Depending on the navigation system accurately mapping the drill to the CBCT image of the jaw used for planning the surgery there will be differences in the accuracy of the osteotomy site preparation (drilling). Drill-tip to CBCT image mapping errors can appear at any point in the mapping chain or because of optical tracking noise or drift away from correct calibration due to thermal, optical and mechanical changes from when the motion system was last calibrated (6,10,15).

In addition, accuracy could be further degraded for any looseness or movement in the rigid coupling between the elements involved. Moreover: motion of patients through the CBCT scanning process, unsTable placement of the jaw attachment, flexing of arms or connectors throughout the surgery and the being tracked movement of the tip of the drill relative to the handpiece (6,8,10,15).

For instance, using a similar navigation system (X-guide, X-nav Technologies) with partially edentulous arches, reported very low *in vitro* mean apex deviations of 0,38 mm/0.89 degrees. In the time *in vivo* studies were four times larger reporting mean deviations of 1,56mm/ 3,62 degrees (6-10,15).

- Comparison with Prior Accuracy studies

The different levels of guidance are influenced by the clinical situation at the time of the surgery (mouth opening, clip stability). The findings among all the different studies are similar, establishing that navigated guidance increases accuracy and precision for implant placement (6).

Block *et al*. [2017] and Vercruyssen *et al*. [2014] report that FH placement was less accurate compared with guided methods. Mean deviations measured with static guides were: 1.6 (0.7) mm at the apex, 1.4 (0.7) mm at entry point (3D) and 3.0 (2.0) degrees for angle deviations versus the corresponding non guided outcomes: 2.9 [1.5] mm, 2.8 [1.5] mm, and 9.9 (6.0) degrees. Concluding higher accuracy in SGS. No statistically significant differences were obtained between the different SGS systems (4-8,10,15).

On one hand D’haese *et al*. [2017] concluded that SGS was associated with fewer errors than real time navigation. And on the other hand Block *et al*. [2017] showed similar accuracy between dynamic and static navigation and superior than non guided implant surgery, as mentioned above. Meanwhile other authors could not find statically significant differences among SGS and DGS (5-16,17).

Nevertheless deviations measured in DGS clinical studies are undoubtedly higher than when *in vitro* models are used. Angular and positional deviations between the planned and actual implant positions were analyzed among the different studies. The following deviations from the virtual plan were evaluated: deviation at the entry point, at the apex and angle discrepancies (6).

The mean deviations over all studies reported were 1.12 (4,5) mm at the entry point, 1.39 [7.1] mm at the apex and 4 (21) degrees for angle discrepancies. Maximal deviation and angle discrepancy were distant from clinically accepTable and feature the risks associated with guide usage. Dissimilar of DGS it is not simple or achievable to evaluate the accuracy of static guidance before or in the course of the osteotomy site preparation. The fact that registration accuracy can be rapidly checked before the first osteotomy by using the drill tip to simply touch visible jaw locations and observing how accurate that location is mapped on the CT image, is an exclusive aspect of DGS. Dynamic navigation permits “on-the-spot” surgical accuracy verification, diminishing the probability of unacceptably large guidance errors (6-12,14,15,17,18).

A reduced number of studies have rated the accuracy among the different dynamic navigation systems, commonly describing an accuracy of 1 to 2mm *in vitro* when using first generation dynamic navigation systems. Stefanelli *et al*. [2019] reported in an *in vivo* study significantly lower accuracy results than the ones described by Somogyi-Ganss *et al*. [2014] earlier in their *in vitro* study (10,16).

Block *et al*. [2017] stated that dynamic navigation will improve in the future accuracy and precision of implant placement, being angulation deviation, the most important parameter improved (6,15).

D’haese *et al*. [2017] reported that DGS in flapless transmucosal interfominal implant placement was precise, predicTable and safe in those patients with a wide, regular and smooth mandibular ridge comparing it with a more irregular bone architecture that ended in a more difficult and less accurate implant placement. Concurrently Stefanelli *et al*. [2019] concluded that no significant differences were obtained between maxillary and mandibular arches or between different mouth sextants (5-10,15).

An additional source of variability is a failure in the process of assessing placement accuracy, potentially affecting the deviations recorded (10,15,16,18,19).

All the authors agree that a learning curve is associated with it, and there’s a need to achieve it. With the use of dynamic navigation a substantial change to exiting clinical workflow and work habits is presented. An up-font investment in equipment, training and skill acquisition is required and may load the practice during the transition and may slow the acceptance of this technology. Improvements in software design and future applications to facilitate the workflow of dynamic navigation surgery may diminish preparation and treatment time for this technology. Despite the fact that the surgical time is similar, or often shorter, employing dynamically navigated implant placement, the overall time for case preparation and planning is higher than freehand surgery (5-16,20-22).

Ongoing research is in progress to erase the need for a pre-made thermoplastic stent over the use of “trace registration mapping” (using skeletal/dental landmarks as references to relate the CBCT image to the patient). These innovation will continue to simplify the workflow and offering at the same time optimal surgical outcomes (5-16,21,22).

## Conclusions

The results of this review suggest that dynamic navigation shows a better accuracy and precision of implant placement (6). The data collected in different studies demonstrate excellent results in terms of accuracy being the experience of the surgeon the single most significant factor on the outcomes (10).

Accuracy resulted to be similar to the reported for static guided surgery and significantly improved when compared to freehand implant placement (15).

Although a true learning curve is associate with it, there have to be improvements to simplify the technique, the workflow and reduce the overall time for case preparation.

To corroborate the results of this review as well as to evaluate the different variables that could influence the accuracy of this technique, future randomized control trials will be needed (8).
